# Antiinflammatory effects of adalimumab, tocilizumab, and steroid on lipopolysaccharide-induced lung injury

**DOI:** 10.3906/sag-2010-303

**Published:** 2021-10-21

**Authors:** Nurhan SARIOĞLU, Fatma Bahar SUNAY, Arzu YAY, Oğuzhan KORKUT, Fuat EREL, Adnan Adil HİŞMİOĞULLARI, Mehmet KÖSE, Betül YALÇIN

**Affiliations:** 1 Department of Pulmonology, Faculty of Medicine, Balıkesir University, Balıkesir Turkey; 2 Department of of Histology and Embryology, Faculty of Medicine, Balıkesir University, Balıkesir Turkey; 3 Department of Histology and Embryology, Faculty of Medicine, Erciyes University, Kayseri Turkey; 4 Department of Pharmacology, Faculty of Medicine, Balıkesir University, Balıkesir Turkey; 5 Department of Biochemistry, Faculty of Medicine, Balıkesir University, Balıkesir Turkey

**Keywords:** Adalimumab, tocilizumab, steroid, acute lung injury

## Abstract

**Background/aim:**

Acute lung injury (ALI) is a major cause of death in the intensive care unit. Lipopolysaccharide (LPS) induced lung injury is the most widely used experimental ALI model and provides opportunities for new targeting therapy. In this study, we investigated the effects of tocilizumab, adalimumab, and methylprednisolone in LPS-induced acute lung injury.

**Materials and methods:**

Lung injury was established by intratracheal instillation of LPS. The rats were randomly divided into six groups: LPS, control, and treatment groups (adalimumab, tocilizumab, methylprednisolone, adalimumab + tocilizumab). Bronchoalveolar lavage (BAL) and lung tissues were collected at 48 h and 96 h following LPS administration from each group. For histological analysis, hematoxylin–eosin (H&E) staining was performed. The sections were obtained for immunohistochemical analysis. IL-6 and TNF-alpha immunoreactivity were measured.

**Results:**

Intratracheal LPS application resulted in inflammatory cell infiltration of interstitial and alveolar spaces and thickening of the alveolar wall. All treatment groups showed signiﬁcantly amelioration compared to LPS at 48 h. Interestingly, adalimumab and adalimumab + tocilizumab groups showed a significant amelioration of the lung histoarchitecture, compared to the prednisolone group at 96 h (p = 0.028, p = 0.025, respectively). Compared to the control group, LPS stimulation resulted in a significant increase in IL-6 and TNF-alpha immunoreactivity (p < 0.001). IL-6 and TNF-alpha expression were markedly reduced in all treatment groups at 48 h but the reduction was greater in the adalimumab and tocilizumab group than in the steroid. Administration with adalimumab and/or tocilizumab effectively decreased expression of TNF-alpha (p = 0.001) and IL-6 (p < 0.001) at 96 h, but prednisolone did not exert an effective decrease (p > 0.05).

**Conclusion:**

Adalimumab and/or tocilizumab significantly reduce the release of proinflammatory cytokines and improve the tissue inflammation in the experimental model of ALI. Our results suggest that adalimumab and/or tocilizumab have a more potent antiinflammatory effect on lung injury than the steroid.

## 1. Introduction

Acute lung injury (ALI) is the most critical illness in the intensive care unit that is characterized by injury of the alveolar capillary and epithelium, interstitial edema, and inflammatory cellular infiltration [1–3]. Inflammation has been known to have a major role in the development of acute lung injury [4,5]. TNF-alpha (TNF-α) and IL-6 are essential proinflammatory cytokines that can induce the production of other cytokines in host response [2,6]. In acute respiratory distress syndrome (ARDS), these cytokines are elevated in both bronchoalveolar lavage (BAL) and plasma [7,8]. The neutrophil gelatinase-associated lipocalin (NGAL) which is the other indicator of inflammation is expressed by neutrophils [9,10]. NGAL is identified as an acute secretary phase protein both in serum and BAL [11].

In the experimental model of ALI, excessive production of proinflammatory cytokines such as TNF-α, IL-1β, IL-6 and NGAL were also detected in BAL [8,11–13]. Previous studies have reported that gram-negative bacterial infection is the main cause of ALI [14–16]. Lipopolysaccharide (LPS), the major component of the cell membrane of gram-negative bacteria, could induce lung injury [17]. In the animal model, administration of LPS with an intratracheal has been shown to cause the production of inflammatory mediators and mimics acute lung injury [18]. 

New targeting molecules focused on these inflammatory cytokines involved the progression of ALI. Some of the most researched agents for early treatment of ALI include corticosteroids, surfactants, statins, nitric oxid, N-acetylcysteine, beta-agonists, vitamin D and C [19,20]. Currently, effective pharmacological treatment has not been developed yet. The use of corticosteroids in ARDS remains controversial [21]. 

Adalimumab, a human monoclonal anti-TNF-α antibody, is commonly used in some inflammatory diseases such as rheumatoid artritis, chron, psoriasis [22]. However, there is not enough knowledge regarding therapeutic effects on lung injury. Tocilizumab, an IL-6R antibody, has been used to treat some cases of COVID-19-related-cytokine storm and ARDS and found beneficial [23]. However, limited data are available on its use in COVID-19 cases and its use in other conditions is not well known [24].

We hypothesis that adalimumab (anti-TNF) and tocilizumab (anti Il-6) can be effective on acute lung injury and ARDS. There are very few animal studies in the literature regarding the use of tocilizumab and adalimumab in lung injury [25–28] and no comparative studies are available. Conflicting results have also been reported in animal studies regarding the use of corticosteroids in acute lung injury [13,29,30]. Some say it has a curative effect on lung injury, while others state that it is unsuccessful [29,30]. 

In this study, we aimed to examine the antiinflammatory effects of tocilizumab and adalimumab in acute lung injury and to assess it by comparing it with the oldest antiinflammatory agent, steroids, whose use in ARDS is controversial.

## 2. Materials and methods

### 2.1. Animals

The Wistar albino male rats (n = 60) (approximately 6 weeks old, weighing 200 to 250 g) were supplied from Uludağ University, Faculty of Veterinary Medicine, Center of Experimental Animals. The rats were kept in plastic cages, the number of the animals in each cage was 4. The room, in which the rats were housed, was supplied with 12 h/12 h light/dark cycle, 22 °C room temperature, and 40%–50% humidity. All animals had free access to pellets for rat and tap water and all of them looked apparently healthy. This study was approved by the local Ethics Committee on animal experiments.

### 2.2. Experimental design

The rats were randomly divided into six groups including 10 rats in each: 1 = LPS, 2 = Control, 3 = LPS + adalimumab (A), 4 = LPS + tocilizumab (T), 5 = LPS + adalimumab + tocilizumab (A+T), and 6 = LPS + prednisolone (P). After anesthetizing the rats with ketamine and xylazine, the trachea was surgically exposed. A total of 20 µg LPS (Escherichia coli, serotype O55:B5 Sigma St. Louis, MO) dissolved in 50 µL of sterile physiological saline was administered intratracheally and lung inflammation was induced in all groups but not the control animals [2,31,32]. The same amount of physiological saline (50 µL) was administered intratracheally to the control group.

Following LPS administration, group 3 (A) received 10 mg/kg adalimumab (anti-TNF-α, Abbott, Wiesbaden, Germany) intraperitoneally (ip) [33], group 4 (T) received 10 mg/kg tocilizumab (anti-IL-6; Roche, Bale, Switzerland) ip [33], group 5 (A+T) received the same doses of the both drugs ip, and group 6 (P) received 5mg/kg methylprednisolone intramuscularly (im) [29]. Control group animals received same volume of sterile physiological saline ip. LPS group animals did not received anything after LPS administration.

Animals in each group divided into two subgroups; 48-h subgroup (n = 5) and 96-h subgroup (n = 5). Animals in 48-h subgroups were sacrificed 48 h after first injection. Animals in 96-h subgroups received a second dose physiological saline, A, T or A+T injection 48 h after the first one, and sacrificed 96 h after their first injections. Animals in P group injected with methylprednisolone every day, and sacrificed 48 h or 96 h after their first injections. Methylprednisolone was administered 5 mg/kg two (n = 5, 48 h) and four days (n = 5, 96 h) in P group. All animals were monitored, namely for general health, appetite, and any sign of infection at the surgical incision site, at least twice on a daily basis until the day they were sacrificed.

BAL was performed by simultaneous injection of 0.6 mL physiological saline three times using a tracheal cannula [31]. The BAL samples were centrifuged at 15,000 rpm for 5 min, and the supernatant was kept at –80 °C until analysis. At the end of the experiment, medial laparotomy and exsanguinations from abdominal artery were applied to rats in all groups, and then the chest cavities were quickly opened and the lungs were removed for histopathological examination.

### 2.3. Histological study

#### 2.3.1. Histopathological evaluation

Harvested lung tissues were fixed in 10% formalin for 24 h, then dehydrated in a graded ethanol series, cleared in xylene, and embedded in paraffin. In addition, 5 μm serial sections were obtained and put on poly-L-lysine slides. All groups were stained with hematoxylin-eosin (H&E) staining to gain a morphological overview of the tissue and its structure. The pathological changes in the lung tissues were visualized under an Olympus BX-51 light microscope (Olympus BX-51, Tokyo, Japan) and photographs were taken. The degree of the lung injury was graded using a scoring system based on the criteria of interstitial inflammation, interstitial damage, leukocyte inﬁltration, intraalveolar hemorrhage, and edema [34]. The scores from 0 to 3 represent the severity: absent = 0, mild = 1, moderate = 2, or severe = 3. The total lung injury score was, then, calculated by adding up the individual scores of each category.

#### 2.3.2. Immunohistochemical analysis 

The sections were deparaffinized in xylene and rehydrated through a graded ethanol series. For immunohistochemical staining, the sections were rinsed in deionized water, and antigen retrieval was performed by incubation in a 10% citrate buffer (pH 6.0) at 95 °C for 5 min, and then cooled to room temperature for 20 min. The sections were incubated in 3% H_2_O_2_ for 10 min, and rinsed in a phosphate-buffered saline (PBS). An antipolyvalent HRP antibody labeling kit (Thermo Scientific, USA) was used for the following steps. To reduce nonspecific staining, the sections were pretreated with normal block serum for 10 min. The sections were, then, incubated with primary antibodies (IL-6 and TNF-α) which were applied to the sections. The slides were, then, incubated overnight at 4 °C in a humidified chamber. The negative control was incubated with a blocking buffer alone rather than with a primary antibody. After washing three times for five min in PBS, the sections were incubated with the biotinylated secondary antibodies for 15 min. After washing in PBS, 3,3 P-diaminobenzidine tetrahydrochloride (DAB) (Lab Vision, UltraVision Detection System Large Volume DAB Substrate System, TA-125-HD) as a chromogen was applied for 3 to 5 min at room temperature. After washing with deionized water, the sections were counterstained with the Gill’s hematoxylin and mounted in a clear mounting medium**. **The stained sections were examined for IL-6 and TNF-α immunoreactivity using an Olympus BX-51 light microscope (Olympus BX-51, Tokyo, Japan).

#### 2.3.3. Quantitative histomorphometry

The quantification of the immunoreactivity intensity of both control and treated-groups were included in the analysis. To evaluate the immunoreactivity intensity of IL-6 and TNF-α, about eight to ten different areas (per visual field) from the experimental groups were randomly selected. The mean immunoreactivity intensity was calculated using the ImageJ software at high power fields (×400 magnification).

### 2.4. TNF-α, IL-6 and NGAL levels in BAL 

BAL samples were centrifuged and the supernatant was collected. All measurements were performed using the NGAL (catalogue no: E-EL-R0662), TNF-α (catalogue no: E-EL-R0019), and IL-6 (catalogue no: E-EL-R0015) enzyme-linked immunosorbent assay (ELISA) kits (Elabscience, USA) according to the manufacturer’s instructions.

### 2.5. Statistical analysis

Shapiro–Wilk and Kolmogorov-Smirnov tests were used to determine whether continuous variables meet the assumption of normality. One way ANOVA was used to compare the averages of the groups if the assumption of normality was met (p > 0.05). Tukey’s test was used as posthoc analysis in comparison to the quantitative data. The results between 48 h and 96 h animals group were evaluated by Mann–Whitney U test. Descriptive data were presented in mean ± standard deviation (SD). Statistical analysis was performed using SPSS version 15.0 software (SPSS Inc., Chicago, IL, USA). p < 0.05 was accepted as statistical significance.

## 3. Results 

### 3.1. Histological findings

**Figure 1:**
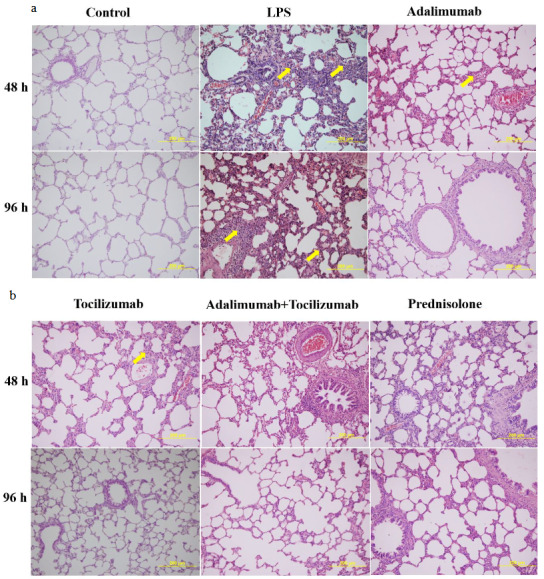
(a and b). Representative micrographs showing the effect of different drugs on the LPS-induced lung histopathological changes. The lung tissues were stained with H&E (original magnification 200×) (Yellow arrow: inflammatory cell infiltration).

We found that LPS-induced pathological changes were signiﬁcantly attenuated by thickening of interalveolar membranes with interstitial edema, inﬂammatory cell inﬁltration, alveolar hemorrhage, and damage of alveoli. However, the lung tissue of the control group showed a normal structure and no evident histological alteration under the light microscope. All treatment groups showed signiﬁcantly amelioration compared to the LPS group (p < 0.001) (Figures 1a and 1b). No significant difference was found among the treatment groups in terms of the histopathological changes at 48 h (p > 0.05) (Figure 2a). However, at 96 h, histological picture of adalimumab (A), adalimumab+ tocilizumab (A+T) groups showed a significant amelioration of the lung tissue compared to the prednisolone (P) group (p = 0.025, p = 0.028, respectively) (Figure 2b). Moreover, the lung tissue architectures of these groups (A, A+T) were close to that in control (p = 0.152, p = 0.497, respectively). 

**Figure 2a:**
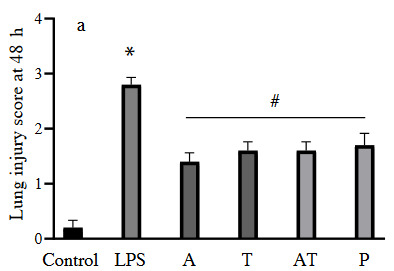
Lung injury score in each experimental group at 48 h (A: adalimumab, T: tocilizumab, P: prednisolone) (*: p < 0.001 vs. control, #: p < 0.05 vs. LPS group).

**Figure 2b:**
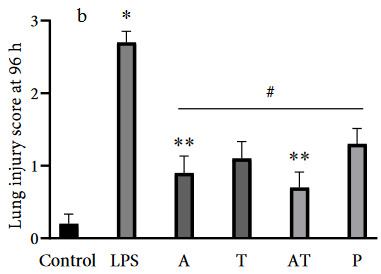
Lung injury score in each experimental group at 96 h (*: p < 0.001 vs. control, **: p < 0.05 vs. prednisolone group, #: p < 0.05 vs. LPS group).

In addition, the findings of each group were compared in itself between 48 h and 96 h. Group A, T, and A+T had significant improvement at 96 h compared to 48 h (p = 0.022, p = 0.023, p < 0.001, respectively), there was no significant improvement in the prednisolone group between 48 h and 96 h (p = 0.094).

### 3.2. TNF-α immunoreactivity intensity

**Figure 3:**
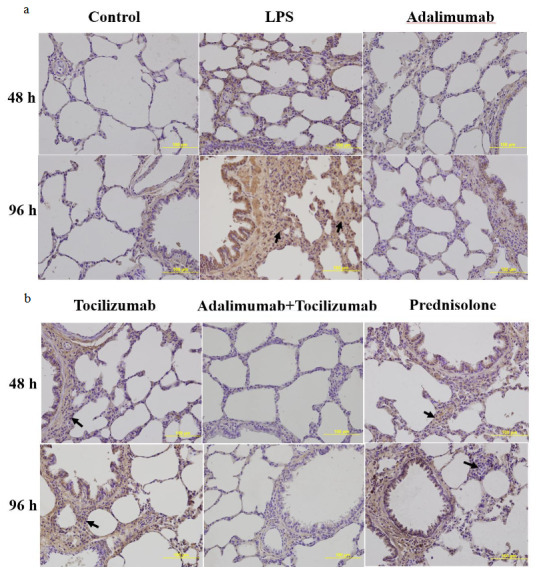
(a and b) Representative micrographs showing the immunoreactivity for TNF-α in tissue sections (×400 magnification) (Arrow: immunoreactivity areas for TNF-α).

In the present study, we assessed the immunoreactivity intensity of TNF-α in each experimental group. The results are shown in Figures 3a and 3b. There was a positive TNF-α expression in all alveolar walls at each experimental group. However, immunoreactivity intensity of TNF-α was weak in the control group, while the administration with LPS significantly increased expression of TNF-α (p < 0.001). At 48 h, TNF-α expression was markedly reduced in all treatment groups (p < 0.001) (Figure 4a). However, the reduction was greater in the group receiving adalimumab and/or tocilizumab than in the steroid group at 48 h (p = 0.001 to p = 0.007) Moreover, group A had the lowest TNF-α immunoreactivity intensity, compared to the other groups, and this level was close to that in the control group (67.5 ± 9.4 vs. 57.3 ± 3.0).

**Figure 4a:**
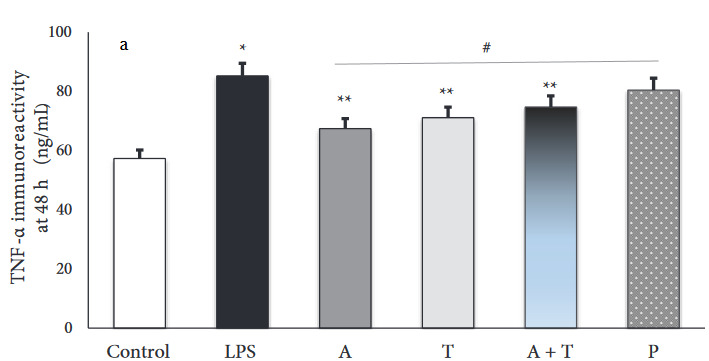
Immunoreactivity intensity of TNF-α in each experimental group at 48 h (*: p < 0.001 vs. control, **: p < 0.05 vs. prednisolone group, #: p < 0.05 vs. LPS group).

**Figure 4b:**
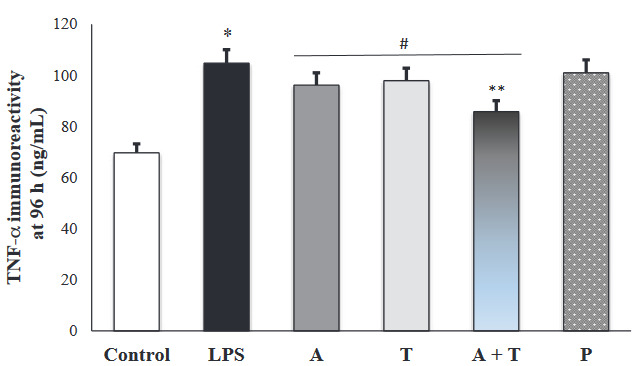
Immunoreactivity intensity of TNF-α in each experimental group at 96 h (*: p < 0.001 vs. control, **: p < 0.05 vs. prednisolone group, #: p < 0.05 vs. LPS group).

Additionally, we shown that adalimumab and tocilizumab effectively reduced TNF-α expression at 96 h (p = 0.001), whereas prednisolone did not significantly (p = 0.409) (Figure 4b).

### 3.3. IL-6 immunoreactivity intensity

**Figure 5:**
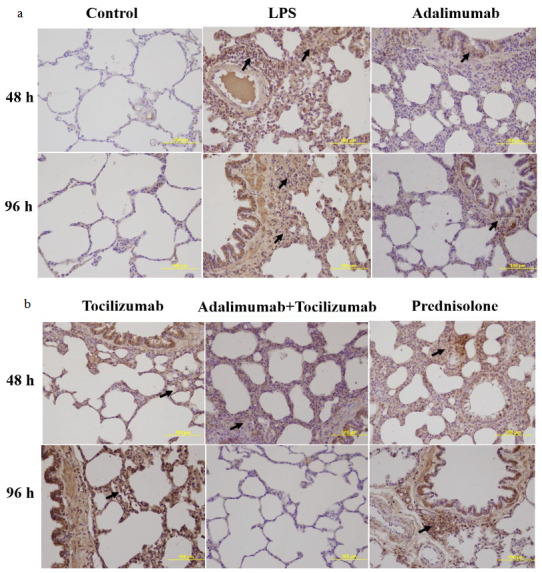
(a and b) Representative micrographs showing the immunoreactivity for IL-6 in tissue sections (×400 magnification) (Arrow: immunoreactivity areas for IL-6).

The results of immunohistochemical staining for IL-6 immunoreactivity are shown in Figures 5a and 5b. Compared to the control group, LPS stimulation significantly increased IL-6 levels at 48 h and 96 h (p < 0.001). At 48 h, IL-6 expression was markedly reduced in all treatment groups (Figure 6a).

**Figure 6a:**
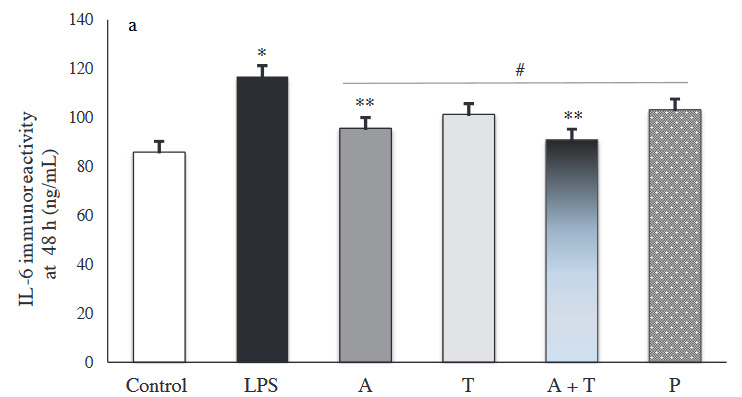
Immunoreactivity intensity of IL-6 in each experimental group at 48 (*: p < 0.001 vs. control, **: p < 0.05 vs. prednisolone group, #: p < 0.05 vs. LPS group).

**Figure 6b:**
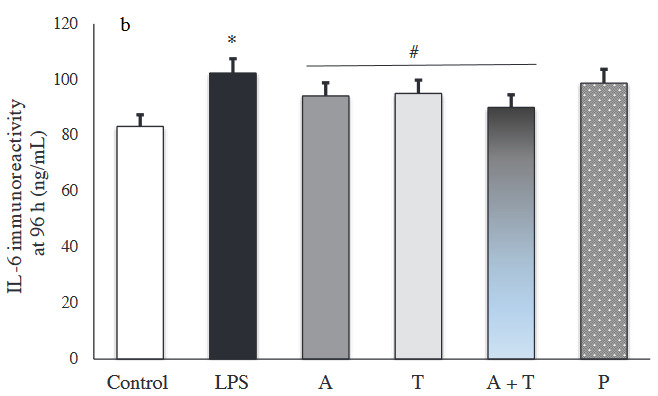
Immunoreactivity intensity of IL-6 in each experimental group at 96 h (*: p < 0.001 vs. control, #: p < 0.05 vs. LPS group).

In particular, group A+T had the lowest IL-6 immunoreactivity intensity, compared to the other groups at 48 h and 96 h. Adalimumab and/or tocilizumab effectively decreased expression of IL-6 at 96 h (p < 0.001), while prednisolone did not exert a significant decrease (p = 0.094) (Figure 6b).

### 3.4. TNF-α, IL-6 and NGAL levels in BAL 

In the present study, TNF-α, IL-6, and NGAL levels in BAL at 48 h were significantly higher in the LPS group than in the control (p = 0.009, p = 0.004, p = 0.032, respectively) (data not shown). TNF-α levels in group A and A+T were decreased compared to LPS but did not statistically significant at 48 h (p = 0.068). IL-6 levels were decreased in all treatment groups but did not statistically significant. NGAL levels in group A were significantly decreased compared to LPS group (p = 0.014) at 48 h. Cytokin levels were decreased in all treatment groups at 96 h but no differences between treatment groups were observed.

## 4. Discussion 

In this study, we investigated the effects of adalimumab, a human monoclonal anti-TNF-α antibody, and tocilizumab (anti-IL-6) on lung inflammation in LPS-induced rats, and compared with steroid treatment. We detected a significant improvement in the lung tissue in each of the treatment groups. However, we observed that adalimumab alone or in combination with tocilizumab resulted in more positive effects on improving than the steroid. Moreover, these two groups (adalimumab and adalimumab+ tocilizumab) were close to that in control concerning lung tissue. 

Inflammation is the major problem that leads to lung injury. The effects of antiinflammatory agents on lung injury remain under investigation. LPS-induced lung injury is the most widely animal model used to mimic ALI [18]. Previous studies indicated that proinflammatory cytokines such as TNF-α, IL-1β and IL-6 are elevated in LPS-induced lung injury [12,13]. In our study, TNF-α and IL-6 expression in the lung tissue increased upon LPS administration. Similarly, we observed that these cytokines and NGAL increased in bronchoalveolar lavage. We demonstrated that administration with adalimumab and/or tocilizumab effectively decreased expression of TNF-α and IL-6, but prednisolone did not exert an effective decrease. These results were consistent with the lung histopathological findings 

In the literature there are some studies about the effect of anti-TNF agents on IL-6 level/expression or vice versa but, the number of studies investigating the effects of these drugs on ALI is very few and as far as we know there is no comparative study [35–37]. Abraham et al. showed that administration of monoclonal anti-TNF-α antibodies reduced the increases in interleukin-1 beta, interleukin-6, and interleukin-10 mRNA levels [35]. Schindler et al. reported that IL-6 treatment to human peripheral blood mononuclear cells suppressed TNF expression at the mRNA level [36]. In a previous study, reciprocal regulation between TNF-α and IL-6 has been shown [37]. In our study we observed that both TNF-α and IL-6 were decreased even when adalimumab and/or tocilizumab were used alone.

Some experimental studies indicate that tocilizumab has a protective effect against acute lung injury [25,26]. It also has been used in some critical patients with COVID-19 and cytokine storm and was found beneficial [38,39]. Results of randomized clinical trials regarding the use of the drug in patients with COVID-19 and ARDS are expected [40]. Tocilizumab holds promise for its use in ARDS cases in the future. In addition to its positive effects, a case of lung opacity due to tocilizumab use as a side effect has also been reported in the literature [41].

There are two animal studies in the literature regarding the use of adalimumab in lung injury: one associated with a ventilator and the other associated with ischemia-reperfusion [27, 28]. As a result of these two studies, it has been shown that adalimumab has a protective effect against lung damage. Similarly, in our study, when adalimumab was given alone or in combination with tocilizumab, it was observed that it had improved effects on lung injury

Anti-TNF agents such as adalimumab are used frequently in many autoimmune and rheumatological diseases [22]. However, it has also been reported in the literature that a patient using adalimumab for hidradenitis suppurativa developed acute lung injury [42]. 

The use of steroids in ARDS is still controversial [21]. Conflicting results have been reported in studies [13,29,30]. Vega et al. reported that dexamethasone has been shown to reduce TNF- α levels in BAL in bleomycin-induced lung injury, but it failed to reduce lung edema and damage scores [30]. Qin et al. showed that dexamethasone reduced TNF- α and IL-6 levels in serum and BAL, and wet/dry weight ratio of lung tissues of rats was decreased [13]. But in that study, histological analysis was not performed, only wet/dry weight ratios of lung tissues were assessed. In another study, methylprednisolone was shown to improve lung damage and inflammation [29].

In our study, both histopathological changes and immunoreactivity for TNF- α and IL-6 were assessed in tissue and measured in BAL. We evaluated the effects of the three different antiinflammatory drugs on lung injury and found that anti-TNF and anti-IL-6 agents were more effective than steroids on lung injury. NGAL was only measured in BAL. LPS stimulation significantly increased NGAL levels and adalimumab effectively decreased. Xiao et al. demonstrated that NGAL mRNA and protein expression levels in the lung tissue and BAL were increased in ventilator‑associated lung injury [9]. More studies are needed to define the main role of NGAL in lung injury.

The limitations of our study are that the results obtained from animal studies cannot be generalized directly to humans. We may have obtained positive results for these drugs in our animal study, but randomized controlled studies are needed to see their effects in humans.

## 5. Conclusion

In the experimental model, based on our study results, adalimumab and tocilizumab were shown to improve lung tissue inflammation and repress the production of proinflammatory cytokines and more effective than steroids.
